# Effects of Dietary Doum Palm Fruit Powder on Growth, Antioxidant Capacity, Immune Response, and Disease Resistance of African Catfish, *Clarias gariepinus* (B.)

**DOI:** 10.3390/ani10081407

**Published:** 2020-08-13

**Authors:** Hanan. S. Al-Khalaifah, Alshimaa A. Khalil, Shimaa A. Amer, Shimaa I. Shalaby, Haitham A. Badr, Mohamed F. M. Farag, Dalia E. Altohamy, Afaf N. Abdel Rahman

**Affiliations:** 1 Environment and Life Sciences Research Center, Kuwait Institute for Scientific Research, P.O. Box 24885, Safat 13109, Kuwait; hkhalifa@kisr.edu.kw; 2 Department of Fish Diseases and Management, Faculty of Veterinary Medicine, Zagazig University, Zagazig 44511, Egypt; shvet2013@gmail.com (A.A.K.); Afne56@gmail.com (A.N.A.R.); 3 Department of Nutrition and Clinical Nutrition, Faculty of Veterinary Medicine, Zagazig University, Zagazig 44511, Egypt; 4 Department of Physiology, Faculty of Veterinary Medicine, Zagazig University, Zagazig 44511, Egypt; siabdallah@zu.edu.eg; 5 Department of Biochemistry, Faculty of Agriculture, Zagazig University, Zagazig 44511, Egypt; egy.hab@gmail.com; 6 Department of Clinical Pathology, Faculty of Veterinary Medicine, Zagazig University, Zagazig 44511, Egypt; farag_cell@yahoo.com; 7 Department of Pharmacology, Central Laboratory, Faculty of Veterinary Medicine, Zagazig University, Zagazig 44511, Egypt; daliaaram1975@gmail.com

**Keywords:** *Hyphaene thebaica*, African catfish, growth, intestinal structure, *Aeromonas hydrophila*

## Abstract

**Simple Summary:**

In aquaculture, medicinal plants and their by-products can be effectively used as natural immune-stimulants, growth promoters, anti-stress agents, and antimicrobials. However, very few studies have been conducted to evaluate the possible use of doum palm fruit powder (DPFP) in diets for African catfish. In this study, four experimental diets with four levels of DPFP (0, 5, 10, and 15 g kg^−1^ diet) in African catfish were evaluated. The findings suggested that DPFP is a good dietary supplement in the aquaculture, which can positively influence fish growth, intestinal histomorphology, hepatic antioxidant activity phagocytic percent and index, lysozyme activity, nitric oxide (NO) production, and disease resistance against *A. hydrophila* challenge.

**Abstract:**

Application of herbal immune-stimulants for modulation of fish growth and immune response has received great interest during the past decades. With several pharmacological properties, Doum palm, *Hyphaene thebaica* (Mart.) is known to be a beneficial medicinal plant. The objective of this study was to investigate the effects of the dietary addition of doum palm fruit powder (DPFP) on growth performance, non-specific immune response, and antioxidant parameters of African catfish, *Clarias gariepinus* (B.). A total of 120 fish (average initial weight 60.50 ± 0.04 g) were randomly allocated to four groups (three replicates/group, 10 fish/aquarium); a basal diet without DPFP supplementation was used as a control, and three other diets were prepared by supplementing 5, 10, or 15 g kg^−1^ DPFP for a ten-week feeding period. Following ten weeks of feeding, the fish were challenged with *Aeromonas hydrophila* (as an immune challenge test), and mortalities were recorded. In comparison to the control diet, dietary DPFP significantly improved growth parameters, including final body weight, body weight gain (WG), specific growth rate (SGR), feed conversion ratio (FCR), and protein efficiency ratio (PER), along with an increase in the content of dry matter of the whole body, in a concentration-dependent manner. Moreover, the heights of intestinal villi, numbers of goblet cells, and intraepithelial lymphocytes (IEL) exhibited marked escalation in all parts of the intestine by increasing the level of DPFP, except for numbers of IEL in the proximal part. The decline in serum glucose, cholesterol, and triglyceride levels was prominent in DPFP10 and DPFP15 groups respective to the DPFP0 group. Furthermore, DPFP boosted the hepatic level of catalase (CAT) in the fish, in a dose-dependent manner; meanwhile, the activity of superoxide dismutase (SOD) and reduced glutathione (GSH) content were also augmented in DPFP10 and DPFP15 groups respective to the DPFP0 group. Dietary DPFP (DPFP15 followed by DPFP10 then DPFP5) led to a pronounced enhancement in the innate immune response (phagocytic percent and index, lysozyme activity, nitric oxide (NO) production, and sialoglycans, namely α 2,3-sialyltransferase and α 2,6-sialyltransferase content); however, the myeloperoxidase (MPO) activity was reduced. Significantly higher relative percentage survival (RPS, 88.56%) of the fish, following the *A. hydrophila* challenge, was observed for the DPFP15 group. We can suggest that DPFP can beneficially influence fish growth, intestinal histomorphology, hepatic levels of catalase (CAT), superoxide dismutase (SOD) activity and glutathione (GSH) content, immune response, and disease resistance against *A. hydrophila* challenge.

## 1. Introduction

As compared to the other food production sectors, the aquaculture industry has flourished significantly in recent times to meet the requirements of the fish market. Fish are considered to be the primary source of nutrition in most poor countries, thereby creating a huge demand [1]. The African catfish, *Clarias gariepinus* (B.), which belongs to the family *Clariidae*, is a widely cultured species in tropical and subtropical countries. Owing to its hardy nature, high cultivability, fast growth rate, reproductive ability in captivity, high survivorship, the capability to accept formulated feed, and somewhat cheaper in comparison with other fish species, African catfish is one of the most preferred fish species for culture in Africa [2,3]. They can dwell a wide range of freshwater habitats like rivers, dams, floodplains, swamps, and lakes. Because of their accessory air-breathing organ, they can survive in hostile environments in turbid, muddy, and oxygen-depleted water bodies [4,5]. Being omnivorous in feeding nature, they can feed on plankton, insects, plants, and snails in the natural water bodies [4].

The fundamental objectives of the fish production industry are to improve fish growth, nutrient digestibility, immunity, and decrease feed cost [6,7,8]. The fish reared under intensive culture, or nutritionally deficient or physiologically unbalanced environments are more susceptible to a wide diversity of bacterial pathogens [9]. These pathogens incur economic losses in the aquaculture industry resulting from mass mortality, cost of treatment, and reduced production [10]. One of these opportunistic pathogens, *Aeromonas hydrophila*, a bacterium, can infect numerous fish species inducing hemorrhagic septicemia [11]. *Aeromonas* infection is usually treated with antibiotics, often developing drug-resistant bacteria and posing a threat to human health [12,13]. Therefore, safe and cheap alternatives are essential to confront these bacterial diseases. The use of plant-based additives in fish diets is regarded as one of these alternatives [7].

Owing to their capability of improving the growth and immunity of fish, herbal feed additives can strengthen the host defense to combat the infectious agents. Henceforth, these herbal additives have grasped the interest of many researchers around the world. However, the roles of different herbal plants in aquaculture need to be elucidated. Various parts of medicinal plants, including leaves, fruits, and roots, have found effective application as functional feed additives in aquaculture [14]. As natural immune-stimulants, to improve health performance [15,16], and as growth promoters [17,18], they are supplemented in the feed in different forms, like powder, oil, or extracts. These immune-stimulating plants can act as an ideal alternative for disease prevention without any hazardous side-effects [19]. 

With its palatable oval fruits, *Hyphaene thebaica* (Mart.) which belongs to the *Arecaceae* family. Doum palm fruit (DPF) has several applications in the food industry, such as in the manufacture of sweetmeats, cakes, and nutritious drinks [20]. The energy required by livestock is obtained from the high protein and carbohydrate (89.25%) contents of DPF [21,22]. Fatty acids, especially the essential linoleic acid and nutritional trace minerals, such as iron, copper, and cobalt, along with low anti-nutritional factors present in this fruit, are vital for the physiological functions of vertebrate organs [23]. Scientists have identified several active constituents of DPF, including flavonoids, polyphenols, saponins, hydroxycinnamates, glycosides, essential oils, and terpenoids. As a consequence, DPF has potent antioxidant, immune-stimulatory, and antimicrobial activities [21]. To avoid the lethal and toxic effects even after DPF overdose, a broad safety margin is essential and the acute toxicity studies of DPF decoction in rats have substantiated adequate safety margin of this fruit [24]. Previous reports have documented the effect of dietary date palm fruit extracts, *Phoenix dactylifera*, a plant in the same family of doum palm, in augmenting growth rate, immune parameters, and antioxidant enzymes activity of common carp, *Cyprinus carpio* [25,26]. Furthermore, Cerezuela et al. [27] and Guardiola et al. [28] reported that the addition of date palm fruit extracts in the diets enhanced the immune response of gilthead sea bream, *Sparus aurata* and European sea bass, *Dicentrarchus labrax,* respectively.

The available information regarding the effect of dietary DPFP on African catfish is very scarce. Therefore, the present study is attempted to evaluate the potential effect of using DPFP as a feed additive on growth, intestinal histology, hematological and biochemical blood parameters, hepatic antioxidant capacity, and immunity augmentation in African catfish.

## 2. Materials and Methods

### 2.1. Preparation of DPFP and Fish Diets

The DPF was bought from a local market at Zagazig city in Egypt. After cleaning the fruits with water to remove any debris, the pulp and seed were separated with a stainless-steel knife. The pulp was dried, crushed, ground into a fine powder with an electric mixer, strained through a 0.25 mm sieve, and finally stored in sealed polyethylene bottles. According to the Association of Analytical Communities, AOAC [29], chemical analysis of the DPFP samples revealed CP (Crude Protein)% (5.83 ± 0.33), ash% (7.66 ± 0.19), fat% (0.86 ± 0.13), crude fiber (CF)% (56.25 ± 0.18), nitrogen free extract (NFE)% (21.4 ± 0.25), and moisture% (8 ± 0.38). As per the recommendations of the National Research Council [30], the basal diet (Table 1) was prepared to satisfy the fish nutrient requirements. The basal diet ingredients were mechanically mixed then pelletized using a meat mincer equipped with a 1.5 mm die. To ensure uniform drying, these pellets were air-dried with regular turning and then were stored at 4 °C in the refrigerator until further use.

### 2.2. Fish Rearing Conditions and Experimental Design

The experimental protocol was approved by the Ethics of the Institutional Animal Care and Use Committee of Zagazig University, Egypt (ZUIACUC-2-F-152-2019).

One hundred and twenty healthy African catfish with an average initial weight of 60.50 ± 0.04 g were obtained from a private fish farm at Abbassa, Sharkia Province, Egypt. No history of outbreaks and no clinical abnormalities were recorded for these fish. According to the norms of CCoA [31], a routine examination of the fish health status was conducted before the experiment. Fish were cultivated in twelve checked static water glass aquaria (80 cm × 40 cm × 30 cm); each was filled with 60 L dechlorinated tap water (10 fish/aquarium) with a daily exchange of water of about 25% with continuous aeration for two weeks. The fish were fed on the basal diet at the rate of 3% of the fish biomass before the initiation of the experiment. According to American Public Health Association (APHA) [32], during the observation period, water parameters were kept within the recommended ranges (dissolved oxygen = 6.18 ± 0.3 mg L^−1^, pH = 7.2 ± 0.5; ammonia = 0.02 ± 0.001 mg L^−1^; nitrite = 0.017 ± 0.001 mg L^−1^; water temperature = 24 ± 2 °C; photoperiod 12:12 light:dark).

The fish were randomly allocated into four groups with three replicates for each (30 fish/group, 10 fish/replicate). The experimental groups comprised of four basal diets with the addition of four doses of DPFP 0, 5, 10, and 15 g kg^−1^ diet (DPFP0, DPFP5, DPFP10, and DPFP15, respectively), for a ten-week feeding period (Adeshina et al., 2020; Amer et al., 2020). Fish were fed 3% of body weight twice daily (8:00 a.m. and 2:00 p.m.). The feed was adjusted every two weeks based on the weight of the fish.

### 2.3. Growth Performance and Survival Rate

The initial weight of the fish was recorded at the start of the experiment; then, the body weights and feed intake were recorded every two weeks, as well as at the end of the experiment. The following growth performance parameters and survival rate were calculated according to Reference [33] as follows:

Weight gain (WG) = Final body weight (g) − Initial body weight (g)

Specific growth rate (SGR) = [(Log_e_ Final weight − Log_e_ Initial weight)/Number of days] × 100

Feed conversion ratio (FCR) = Feed given (Dry weight)/Body WG (Wet weight)

The protein efficiency ratio (PER) = Net WG (Wet weight)/Protein fed

Survival rate (SR) = Total number of fishes harvested/Total number of fishes stocked × 100.

### 2.4. Proximate Whole Body Composition Analysis

At the end of the feeding trial, three fish from each group were randomly selected for the chemical analysis of the fish body, according to AOAC [29]. Fish samples were frozen at −20 °C till analyzed. Determination of moisture, crude protein, ether extract, and ash content was conducted by thawing the frozen whole fish, drying in the hot air oven, blending, and then analyzing. To estimate the moisture content, the samples were dried in a drying oven (GCA, model 18 EM, Precision Scientific Group, Chicago, IL, USA) at 85 °C until a constant weight is achieved. The crude protein (N × 6.25) was determined using the Kjeldahl distillation unit (UDK 129, VELPScientifica, UsmateVelate, Via Stazione, Italy), whereas Soxhlet extractor glassware with petroleum ether (60–80 °C) was used to estimate crude lipids. The ash content was quantified using muffle furnace (Barnstead/Thermolyne Benchtop 47900, Thermo Scientific, Waltham, MA, USA).

### 2.5. Sampling

At the end of the experiment, three fish from each aquarium (9 fish/group) were randomly chosen and anesthetized using 95 mg L^−1^ clove oil (Oleum, Cairo, Egypt) within 3 min [34]. Thereafter, blood samples were collected from the caudal vein in two aliquots. The first aliquot was collected with the help of sterile heparinized syringes for measuring hematological indices and phagocytic activity. The second aliquot, collected without anticoagulant, was centrifuged at 1075× *g* for 20 min for serum separation for measuring the biochemical and immunological indices. Furthermore, for the evaluation of the antioxidant enzyme activity, liver samples were isolated and frozen at −20 °C until analysis. Samples of the intestine were also removed and preserved in 4% buffered formalin for histological examination.

### 2.6. Intestinal Morphometric Analysis

At the end of the experiment, approximately 1 cm-long piece of the three intestinal parts (proximal, middle, and distal) was fixed in 4% buffered formalin. The specimens were dehydrated using ascending grades of ethyl alcohol (70–100%), cleared in xylene, and embedded in paraffin. With the help of microtome (Leica^®^, Wetzlar, Germany), tissue sections of 5 µm thicknesses were prepared and stained with hematoxylin and eosin (H&E). Slides were visualized for morphometric analysis and photographed using the AmScope digital camera-attached Ceti England microscope for histopathological examination [35]. For morphometric analyses, 20 images per animal were captured at 40× and 400× for villous height and Goblet cells and intraepithelial lymphocytes (IEL) counting was conducted using AmscopeToupView 3.7 software (AmScope, Irvine, CA, USA). The intestinal villous heights were measured from the tip of the villous to its base in all parts of the intestine. The number of goblet cells and IEL were counted, according to Pirarat et al. [36].

### 2.7. Hematological and Biochemical Assays

An automated hematology analyzer (Hospitex Diagnostics, Sesto Fiorentino, Italy) was used to count the total erythrocyte (RBC) and leukocyte (WBC), following the method described by Feldman et al. [37]. According to the methods described by Jain [38], the hemoglobin (Hb) and hematocrit (Ht) concentrations were quantified immediately after sampling.

With the help of the colorimetric diagnostic kits of spectrum-bioscience (Egyptian Company for Biotechnology, Cairo, Egypt), total cholesterol, triglycerides, and glucose were estimated following the protocols of Allain et al. [39], McGowan et al. [40], and Trinder [41], respectively. The qualitative fractionation of serum proteins using cellulose-acetate electrophoresis was done according to Kaplan and Savory [42].

### 2.8. Hepatic Antioxidant Capacity Assays

The activities of catalase (CAT) and superoxide dismutase (SOD) enzymes and the reduced glutathione content (GSH) of the liver samples from three fish from each replicate (9 in total/group) were assessed using the kits from Biodiagnostic Co. (Cairo, Egypt) according to the methods described by Sinha [43], McCord and Fridovich [44], and Patterson [45], respectively.

### 2.9. The Phagocytic Activity

The phagocytic activity (%) of leucocytes was assayed, and phagocytic percent and phagocytic index were calculated according to the formula of Siwicki et al. [46]:

Phagocytic activity% = (No. of phagocytic cell phagocytizing bacteria/Total no. of phagocytic cells counted) ×100

Phagocytic index = Total no. of phagocytized bacteria/No. of phagocytic cells phagocytizing bacteria.

### 2.10. The Serum Lysozyme and Myeloperoxidase Activity and Nitric Oxide Level

According to the method of Grinde [47], the lysozyme activity was assayed using the lysoplate technique. The activity of myeloperoxidase (MPO) and nitric oxide (NO) level were measured according to the previously described methods of Quade and Roth [48] and Moshage [49], respectively.

### 2.11. Lectin Solid-Phase Immunoassay

The α 2,3-sialyltransferase (α 2,3-ST) and α 2,6-sialyltransferase (α 2,6-ST) activities to galactose were estimated by lectins in a sandwich solid phase assay Kletter et al. [50].

### 2.12. Challenge Test

At the end of the experiment, all experimental groups were inoculated with the pathogenic bacterium *A. hydrophila* at a 0.1 mL dose of suspension cell containing 1.5 × 10^7^ mL^−1^ cells using standard McFarland tubes through intraperitoneal injection. *A. hydrophila* was previously isolated from the dying fish and confirmed to be pathogenic for African catfish by the Department of Fish Diseases and Management of the Faculty of Veterinary Medicine at Zagazig University. *A. hydrophila* was identified by conventional biochemical tests and an automated VITEK 2-C15 system for bacterial identification (BioMérieux, Marcy-l’Étoile France) according to the manufacturer’s instructions at the Department of Microbiology and Immunology, National Research Center (NRC), Dokki, Giza, Egypt as explained by Reference [51,52]. According to Lucky [53], fish mortalities were recorded for two weeks and were used for calculating the relative percentage survival “RPS” based on Amend [54].

### 2.13. Statistical Analysis

All data were expressed as means ± standard error (SE). All data were verified for normality after transformation (ASIN). ANOVA (Analysis of Variance) test was used based on polynomial orthogonal contrasts. Linear and quadratic regression equations were performed using SPSS (Statistical Package for Social Sciences) Version 17 for Windows (SPSS Inc., Chicago, IL, USA) on growth, intestinal morphometric, biochemical, hematological, antioxidant, and immune parameters to evaluate the relationship between DPFP levels and these parameters at a significance value of *p* < 0.05. Post-hoc Tukey’s test was applied to determine differences among means based on linear regression. The statistical significance was based on *p* < 0.05 unless otherwise stated.

## 3. Results

### 3.1. Growth Performance and SR

The growth performance and SR of African catfish fed on diets enriched with DPFP is shown in Table 2. The dietary DPFP (DPFP5, DPFP10, and DPFP15) consumption linearly and quadratically increased the final body weight (1.15-, 1.24-, and 1.26-fold; *p* = 0.00, *p* = 0.002), total WG (1.35-, 1.54-, and 1.59-fold; *p* = 0.001, *p* = 0.00), total feed intake (1.192-, 1.194-, and 1.22-fold; *p* = 0.00, *p* = 0.001), SGR (1.25-, 1.36-, and 1.39-fold; *p* = 0.00, *p* = 0.001), and PER (1.31-, 1.48-, and 1.49-fold; *p* = 0.00, *p* = 0.00), and decreased the FCR (0.88-, 0.77-, and 0.76-fold; *p* = 0.001, *p* = 0.01), respectively, of fish as compared to that of the control group (DPFP0). However, the effect of DPFP on African catfish SR was found to be statistically insignificant.

### 3.2. The Proximate Whole-Body Composition

The proximate whole body composition analysis is presented in Table 3. As compared to the control group, the fish fed on DPFP10 and DPFP15-supplemented diets followed by DPFP5 recorded higher linear values for the body content from the dry matter (1.18-, 1.35-, and 1.12-fold; *p* = 0.001), respectively. Moreover, the crude protein content increased linearly and quadratically (1.91- and 2.33-fold; *p* = 0.003, *p* = 0.004), whereas the ash content increased linearly (1.75- and 2.13-fold; *p* = 0.005) in DPFP10 and DPFP15 groups, respectively, compared to the control group. A linear elevation in crude lipids (1.11-fold; *p* = 0.005) was also perceived in the DPFP15 group relative to the control group.

### 3.3. Morphometric Measures of the Intestine

**Figure 1:**
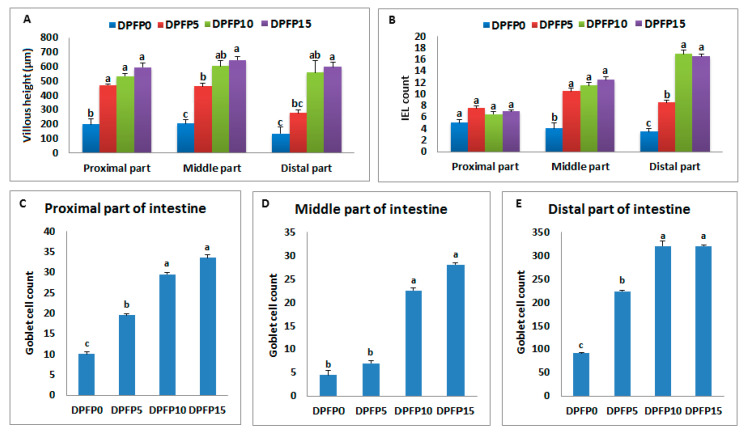
The average villous height (µm) (**A**), intraepithelial lymphocytes count, intraepithelial lymphocytes (IEL) (**B**), and goblet cell count (**C**–**E**) of intestinal parts (proximal, middle, and distal) of African catfish fed on DPFP-enriched diets at 0, 5, 10, and 15 g kg^−1^ (DPFP0, DPFP5, DPFP10, and DPFP15), respectively, for 10 weeks. The groups with different superscript differ significantly (*p* < 0.05). Data were expressed as Mean ± SE, n = 3.

As shown in Figure 1A, significant elevation of the heights of intestinal villi in all parts of the intestine was evident by increasing the dose of DPFP. The heights were found to increase linearly and quadratically (2.35-, 2.66-, and 2.97-fold; *p* = 0.00, *p* = 0.01) in the proximal and (2.26-, 2.96-, and 3.17-fold; *p* = 0.002) middle parts, whereas only linearly (2.07-, 4.18-, and 4.47-fold; *p* = 0.00, *p* = 0.003) in the distal part of the intestine in DPFP5, DPFP10, and DPFP15-fed groups, respectively, relative to the control group.

No significant effect of the DPFP on the IEL number in the proximal part of the intestine (Figure 1B) was witnessed in the present study. Meanwhile, compared to the control group (DPFP0), linear and quadratic dose-dependent increase in their number was documented in the DPFP-fed groups (DPFP5, DPFP10, and DPFP15) in the middle (2.62-, 2.87-, and 3.12-fold; *p* = 0.001, *p* = 0.01) and distal parts (2.43-, 4.86-, and 4.71-fold; *p* = 0.00, *p* = 0.01), respectively (Figure 1).

The count of goblet cells depicted higher linear and quadratic increase in the proximal (1.95-, 2.95-, and 3.35-fold; *p* = 0.00, *p* = 0.04) (Figure 1C) and distal parts (2.44-, 3.50-, and 3.50-fold; *p* = 0.00, *p* = 0.01) (Figure 1E) of DPFP5, DPFP10, and DPFP15 groups, respectively, than the DPFP0. The middle part (*p* = 0.001) of the intestine also reflected a linear increase in the count of the goblet cells for the group fed on DPFP10 (5-fold) and DPFP15 (6.22-fold) diets compared to the control fish (Figure 1D).

### 3.4. Histological Outcomes

**Figure 2:**
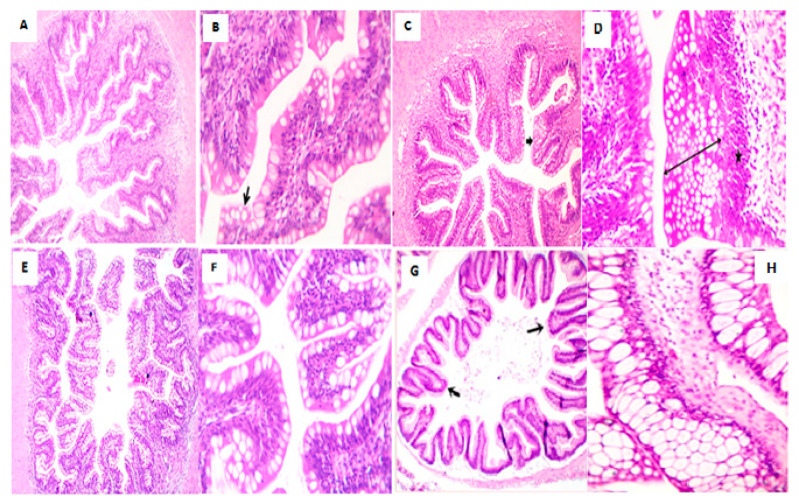
Photomicrograph of the posterior part of the fish intestine (**A**) showed tall and serrated villi surfaces with mildly thickened lamina propria. Hematoxylin and eosin (H&E) ×100 (**B**) showed moderate goblet cell metaplasia (arrow) in serrated villi surface with mild intraepithelial and lamina propria lymphocytic infiltration. H&E ×400 (**C**) showed thickly branched villi with marked goblet cells metaplasia (arrow). H&E ×100 (**D**) showed large goblet cells at several rows (two-sided arrow) followed by proliferated enterocytes (star). H&E ×400 (**E**) showed marked short thick villi with goblet cells metaplasia. H&E ×100 (**F**) showed intense goblet cells metaplasia and lymphocytic infiltrations. H&E ×400 (**G**,**H**) showed tall villi with numerous broad tips (arrows) and goblet cell metaplasia at low 40× and high magnification 400×. (DPFP0 = A,B; DPFP5 = C,D; DPFP10 = E,F; DPFP15 = G,H).

Histological sections of the posterior part of fish intestine illustrated- moderate goblet cell metaplasia in tall and serrated villi surfaces with mild intraepithelial and lamina propria lymphocytic infiltration in DPFP0 group; thickly branched villi with marked goblet cells metaplasia at several rows and proliferated enterocytes in DPFP5 group; short thick villi with goblet cells metaplasia and lymphocytic infiltrations in DPFP10 group; tall villi with numerous broad tips and goblet cell metaplasia in DPFP15 group (Figure 2).

### 3.5. Hematological and Biochemical Indices

The results, represented in Table 4, showed a linear and quadratic increase in the RBC count (1.48-, 1.66-, and 1.70-fold; *p* = 0.00, *p* = 0.001), WBC count (3.06-, 3.82-, and 4.18-fold; *p* = 0.00, *p* = 0.01), and Hb (1.47-, 1.55-, and 1.63-fold; *p* = 0.00, *p* = 0.003) content, and only a linear increase in the HT% (1.40-, 1.75-, and 1.94-fold; *p* = 0.002) was associated with a concentration-dependent increase in the DPFP level (DPFP5, DPFP10, and DPFP15), respectively, as compared to the DPFP0 group.

With regards to the control (DPFP0) group, a linear decline in the levels of glucose (0.91 and 0.88-fold; *p* = 0.002) and triglyceride (0.71 and 0.68-fold; *p* = 0.00) and linear and quadratic decreases in the cholesterol level (0.88 and 0.75-fold; *p* = 0.001, *p* = 0.01) were noted in DPFP10 and DPFP15 groups, respectively (Table 4).

The serum total protein, as demonstrated in Table 4, was linearly enhanced (*p* = 0.00) in DPFP10 (1.35-fold) and DPFP15 (1.49-fold) groups with respect to the DPFP0 group. Linear elevation was also obtained in the serum total globulin (1.36-, 1.63-, and 1.86-fold; *p* = 0.004) and its fraction γ globulin (3.19-, 4.72-, and 5.05-fold; *p* = 0.001) in DPFP-fed groups (DPFP5, DPFP10, and DPFP15), respectively, as compared to the control group. However, DPFP failed to illuminate any significant effect on serum albumin and other globulin fractions.

### 3.6. Hepatic Antioxidant Capacity

As shown in Table 5, the CAT activity of African catfish (1.55-, 1.82-, and 2.16-fold, *p* = 0.001) was linearly augmented by the DPFP-supplemented diets (DPFP5, DPFP10, and DPFP15) respectively, higher than the DPFP0 group. Compared to the control group, there was a linear increase in the SOD activity (4.22- and 4.87-fold; *p* = 0.001) and GSH content (2.13, and 2.94-fold; *p* = 0.00) in fish fed DPFP10 and DPFP15, respectively.

### 3.7. Immune Parameters

Table 5 illustrated DPFP-supplemented diet (DPFP5, DPFP10, and DPFP15) was responsible for a linear increase in the phagocytic (%) (1.19-, 1.27-, and 1.45-fold; *p* = 0.003), phagocytic index (1.40-, 1.67-, and 1.91-fold; *p* = 0.004), and lysozyme activity (1.62-, 2.10-, and 2.71-fold; *p* = 0.001) and decrease in MPO activity (0.84-, 0.64-, and 0.56-fold; *p* = 0.00), respectively, as compared to the DPFP0 group.

Regarding NO activity (Table 5), there were linear and quadratic enhancements (*p* = 0.001, *p* = 0.01) observed in fish fed DPFP, where NO activity was (1.43-, 1.76-, and 1.88-fold higher than the control group) in DPFP5, DPFP10, and DPFP15 groups, respectively. DPFP-supplemented diets could linearly augment the α 2, 3-ST (11.60-, 13.40-, and 15-fold; *p* = 0.02) and α 2, 6-ST (4.93-, 6.57-, and 7-fold; *p* = 0.01) activity, respectively, more than the control diet (Table 5).

### 3.8. Challenge with A. hydrophila

The mortality rate, survival (%), and RPS of the African catfish, fed on DPFP-enriched diets for ten weeks and challenged with *A. hydrophila*, were recorded. This would reflect the resistance of the fish against the pathogen. The highest mortality rate was recorded in the control group (66.67%), followed by those fed on DPFP5 (44.45%) and DPFP10 (22.22%), whereas the lowest mortality rate was recorded in those fed on DPFP15 (7.63%). An increase in the concentration of the DPFP was associated with an enhanced survival rate in the fish, where it was 55.55%, 77.78%, and 92.37% in DPFP5, DPFP10, and DPFP15, respectively, as compared with the control group (33.33%). The RPS was found to be highest in the DPFP15 group (88.56%), followed by the DPFP10 group (66.67%), whereas it was lowest in the DPFP5 group (33.33%).

## 4. Discussion

Dietary DPFP was responsible for a concentration-dependent increase in the growth performance, feed efficiency, and utilization of African catfish in the present study. Although there is no available data on the impact of doum palm on fish growth and feed intake, the growth improvement offered by dietary doum palm can be returned to better nutritional status. High contents of proteins (2.86–5.01%), fibers (52.26–66.5%), essential fatty acids, vitamins, and minerals in doum palm justify its high nutritional value [21,23]. Moreover, the mesocarp of doum palm is very palatable and highly aromatic with a sweet smell [55]. The sweet smell of its fat act as an attractant for consumption [56], motivating appetite and enhancing feed intake. The present study findings were in accordance with the results of [26] on common carp. They highlighted that consumption of the dietary date palm fruit extract of 200 mL kg^−1^ for eight weeks could induce growth-related gene expression and improve fish weight by 34.6% and feed utilization by 17.53%.

Furthermore, the present study also established the effect of dietary DPFP in augmenting the villous heights and numbers of goblet cells and IEL in all parts of the intestine except count of IEL in the proximal part. Increased surface area for the absorption, improved feed digestion, nutrient utilization, and defense against pathogens by mucin layers on the intestinal mucosa were, therefore, substantiated by these findings, which in turn reflected the effect on the fish growth performance as reported by Reference [57]. The digestive and absorptive capacity of the fish is estimated by the effective indicators, including the numbers of goblet cells (mucus-secreting cells) and the intestinal villous heights [16,58,59]. Defense against foreign antigens is attributed to IEL, effective components of gut-associated lymphoid tissue [60]. The high IEL count in DPFP-fed fish validated the increased intestinal immune cells; however, in-depth research is required to uncover the mechanisms behind this elevation. Histomorphological study of the intestine revealed similar results in African catfish fed on diets supplemented with Indian lotus, *Nelumbo nucifera* stamen extract [61]. Abdel-Tawwab et al. [62] also observed improvement in the intestinal structure of African catfish resulting from dietary incorporation of clove basil, *Ocimum gratissimum* leaves extract for 12 weeks.

The dry matter of African catfish was redundant with increasing the level of DPFP. An increase in the crude protein and ash content was reported in the DPFP10 and DPFP15 groups; however, marked elevation of the crude lipids was witnessed in the DPFP15 group. Enhanced feed efficiency and utilization by DPFP, as well as improved digestibility, might be held responsible for these findings as the carcass composition has been demonstrated to be influenced by the diet [63]. As reported by Nwosu et al. [56], the increased body ash content can be attributed to the higher ash content of DPFP (8.10 ± 0.06%). A similar increase in body protein content (20% on wet basis) has been documented in African catfish fed on diets enriched with Ugu, *Telfera occidentales* leaves [64].

The hematological indices are used as biomarkers to evaluate the physiological condition and health status of fish due to the influence of immunostimulants [65,66,67]. The DPFP-fed fish exhibited significant enhancements in the RBC and WBC count, Hb content in this study. The phytochemicals present in DPFP, such as flavonoids, reducing sugars, glycosides, and some minerals, such as iron, cobalt, and copper, may contribute to these effects by stimulating the synthesis and maturation of blood cells [68,69]. These findings, therefore, indicated that DPFP could promote the synthesis of RBCs, WBCs, and other blood elements in the hematopoietic tissues, resulting in the immune-modulatory effect and increased disease resistance, as WBCs are known to be the vital players of the innate immune system [70]. Parallel works in African catfish fed on diets supplemented with Aloe vera [71] and pawpaw or bitter gourd, *Vernonia amygdalina*, or their combination [72] have also been published.

The primary source of energy for fish to withstand unfavorable conditions is blood glucose, which is also an efficient indicator of stress. Cholesterol and triglycerides are the lipids present in the blood in the form of lipoproteins. Among the several factors that influence the concentrations of cholesterol and triglycerides, diet formulation is the most important one [72]. The present study illustrated the effect of dietary DPFP in reducing serum glucose, cholesterol, and triglycerides levels of African catfish. The increased pancreatic secretion of insulin or the enhanced peripheral metabolism of glucose-induced by DPFP may be responsible for the low glucose level [73]. Pectins present in DPFP may lower the total cholesterol by reducing the cholesterol absorption from the diet and, therefore, may be attributed to the cholesterol and triglyceride-lowering effect of DPFP [74]. The lipid-lowering effect of DPFP can also be explained by its high content of antioxidants, such as flavonoids and polyphenols compounds, that prevent lipid peroxidation and formation of lipo-peroxides, which are atherosclerosis agents [21,75]. Moreover, glycosides (saponins) present in doum palm are known to form complexes with cholesterol and bile in the intestine, thereby indirectly reducing the cholesterol level in the blood, which may also rationalize this effect of DPFP [24]. Likewise, dietary clove basil leaf extract [62] and Aloe vera polysaccharides [76] have also reported such lipid-lowering activity in African catfish.

Elevated levels of blood proteins, especially globulin, are good indicators of the increased immune response [77]. DPFP induced marked escalation of the serum total protein, total globulin, and its fraction gamma globulin values have been demonstrated in the present study findings. The potential of DPFP to increase defensive proteins are validated by these results, which further indicate the immune-modulatory activity of this plant-based on its phytochemical constituents. Following these outcomes, African catfish fed on diets supplemented with ginger, *Zingiber officinale* and Roselle, *Hibiscus Sabdarifa* for 70 days have also documented a parallel increase in the protein profile [78]. Furthermore, research findings published by Reference [62] also illuminated the elevation of the total protein levels in African catfish fed with clove basil.

Oxidative stress is detrimental to vital biomolecules, such as DNA and proteins. This oxidative damage of the body tissues is prevented by the antioxidant system that eradicates the reactive oxygen species [79]. Dietary antioxidants aggravate the free radical scavenging ability of these enzymes [15,19,62]. Present study findings confirmed the effect of DPFP in improving the antioxidant enzyme status. Flavonoids and polyphenols are known to scavenge oxygen free radicals and prevent the fatty acids lipo-peroxidation in the cell membrane and cellular oxidation. High levels of these phytochemicals in DPF may, therefore, contribute to the antioxidant properties of DPF [75,80,81]. Similar results have been recorded regarding the effect of dietary date palm fruit extracts on antioxidant enzyme system of gilthead seabream [27,82], European sea bass [28], and common carp [25].

In contrast to mammals, innate immunity is the major player of defense in the case of fish. Phagocytic cells (neutrophils and macrophages) are important components of the innate immunity, which obliterate the pathogens through a process called phagocytosis [83]. Moreover, to stimulate the ability of phagocytes to kill pathogens, NO, a strongly reactive oxygen molecule, is released by the macrophages [84]. Initiation of phagocytosis involves the activity of another component- lysozyme, produced by leucocytes. Bactericidal action of the lysozyme is mediated via the lysis of the bacterial cell wall [85]. A major post-translational modification of proteins- glycosylation- is associated with various biological processes, including immune response [86]. In this study, dietary DPFP was found to enhance the non-specific immunological defenses (phagocytic activity, lysozyme, NO, and serum bound α 2,3-ST and α 2,6-ST). These findings also indicated that DPFP increased sialoglycans and a higher degree of glycosylation states by entrapping terminal sialic acid moieties, included in the glycoproteins as reported by Coombe and Parish [87]. As reported by Auwal et al. [70], the bioactive components of DPFP, including flavonoids, glucosides, such as saponins, terpenes, and others, are known to exert immunomodulatory activity and may, therefore, be correlated to these actions of DPFP. Similar outcomes have been observed in common carp [25,26], European seabass [28], and gilthead seabream [27] fed with dietary date palm.

Activated neutrophils release the enzyme MPO, a well-known heme-containing peroxidase. The killing of pathogens is a crucial role of this enzyme, and it also acts as a local mediator of tissue damage resulting in oxidative stress and inflammation [88]. Present study perceived reduced serum MPO activity in response to DPFP. Phytochemicals, like flavonoids, coumarins, sphingolipids, and oxygenated fatty acids, act against oxidative damage or inhibit cyclooxygenase, an enzyme involved in inflammation [89]. The high content of these phytochemicals may correlate these findings with the anti-inflammatory effect of DPFP.

The challenge test is mostly used as a confirmatory assay to measure the organism’s immune functions as a whole [90]. The efficacy of immuno-stimulants can be conclusively validated by the improved resistance of fish against infectious agents [91]. The study showed DPFP diets induced remarkable improvement of the survival rate of the fish against *A. hydrophila* pathogen. The beneficial effects of DPFP on the survival rate of African catfish might be attributed to its active compounds that could enhance the non-specific immune parameters like phagocytic activity, NO, lysozyme, sialoglycans, and antioxidant enzymes and its antimicrobial activity. According to Olusola and Nwokike [72] and Abdel Rahman et al. [19], herbal immune-stimulants are effective in enhancing the resistance of African catfish to *A. hydrophila*.

## 5. Conclusions

The present results established the beneficial effects of DPFP on fish growth, intestinal histomorphology, hepatic antioxidant activity represented by increased catalase, superoxide dismutase activity, and glutathione content, immune response represented by increased phagocytic percent and index, lysozyme activity, nitric oxide production, sialoglycans content, and reduced myeloperoxidase activity, and improving disease resistance of African catfish against *A. hydrophila* challenge. Further studies are encouraged to elucidate its potential applications on the growth and health of other fishes.

## Figures and Tables

**Table 1:** Feed formulation and proximate composition (g kg^−1^ on a dry weight basis).

Ingredients	g kg^−1^
Yellow corn	213.5
Wheat flour	100
Soybean meal 49% CP	310
Corn gluten 67% CP	100
Fish meal 70.7% CP	150
Wheat bran	60
Fish oil	60
Methionine	3.5
Vitamins and minerals mixture ^1^	3
Proximate composition (g kg^−1^) ^2^	
Crude protein	371.6
Fat	96.1
Crude fiber	39.2
NFE ^2^	431.3
Ash	61.6
Lysine	20.04
Methionine	10.8
GE KJ/kg ^3^	207.2

^1^ Composition of vitamins and minerals premix kg^−1^: vitamin A 580,000 IU; vitamin D_3_ 8600 IU; vitamin E 720 mg; vitamin K_3_ 142 mg; vitamin C 0.1 mg; vitamin B_1_ 58 mg; vitamin B_2_ 34 mg; vitamin B_6_ 34 mg; vitamin B_12_ 58 mg; biotin 50 mg; folic acid 86 mg; pantothenic acid 8 mg; manganese sulfate 65 mg; zinc methionine 3000 mg; iron sulfate 2000 mg; copper sulfate 3400 mg; cobalt sulfate 572 mg; sodium selenite 25 mg; calcium iodide 25 mg; calcium carbonate as carrier up to till 1 kg. ^2^ Nitrogen free extract, determined by difference = 100 − (protein% + fat% + crude fiber% + ash%). ^3^ Gross energy (GE) was calculated according to National Research Center (NRC) (2011) as 23.6 KJ/g protein, 39.5 KJ/g lipid and 17.0 KJ/g NFE.

**Table 2:** The effects of dietary doum palm fruit powder (DPFP) on the growth performance and survival rate of African catfish.

Parameters	DPFP0	DPFP5	DPFP10	DPFP15	Regression Analysis ^#^	
					Linear	Quadratic
IBW/fish (g)	60.56 ± 0.09 ^a^	60.47 ± 0.11 ^a^	60.46 ± 0.05 ^a^	60.53 ± 0.09 ^a^	0.78	0.39
FBW/fish (g)	109.64 ± 0.74 ^c^	126.91 ± 1.17 ^b^	136.09 ± 0.84 ^a^	138.65 ± 1.69 ^a^	0.00	0.002
Total BWG/fish (g)	49.08 ± 0.67 ^c^	66.44 ± 1.28 ^b^	75.63 ± 0.80 ^a^	78.12 ± 1.78 ^a^	0.001	0.00
Total FI/fish (g)	125.88 ± 0.78 ^c^	150.00 ± 0.23 ^b^	150.30 ± 0.09 ^ab^	153.68 ± 1.33 ^a^	0.00	0.001
SGR (%/day)	0.85 ± 0.01 ^c^	1.06 ± 0.02 ^b^	1.16 ± 0.01 ^a^	1.18 ± 0.024 ^a^	0.00	0.001
FCR	2.57 ± 0.11 ^a^	2.26 ± 0.12 ^b^	1.99 ± 0.05 ^c^	1.97 ± 0.124 ^c^	0.00	0.001
PER	1.17 ± 0.01 ^c^	1.53 ± 0.03 ^b^	1.74 ± 0.01 ^a^	1.75 ± 0.04 ^a^	0.00	0.00
Survival rate (%)	100 ± 0.00	100 ± 0.00	100 ± 0.00	100 ± 0.00	0.45	0.63

^#^ The regressions were considered significant at *p* < 0.05. IBW, Initial body weight; FBW, Final body weight; BWG, Bodyweight gain; FI, feed intake; SGR, Specific growth rate; FCR, Feed conversion ratio; PER, Protein efficiency ratio. Data were expressed as Mean ± SE, n = 3; ^a,b,c^ Mean values in the same row with different superscripts differ significantly (*p* < 0.05).

**Table 3:** The effects of dietary doum palm fruit powder (DPFP) on the proximate whole-body composition of African catfish.

Parameters	Initial	DPFP0	DPFP5	DPFP10	DPFP15	Regression Analysis ^#^	
						Linear	Quadratic
DM *	20.70 ± 0.11	23.50 ± 0.17 ^c^	26.35 ± 0.51 ^b^	27.77 ± 1.16 ^a,b^	31.74 ± 1.81 ^a^	0.001	0.70
Crude protein **	58.83 ± 0.07	60.48 ± 0.27 ^b^	61.25 ± 0.25 ^a,b^	62.39 ± 0.11 ^a^	62.81 ± 0.19 ^a^	0.003	0.004
Crude lipids **	10.64 ± 0.28	12.40 ± 0.10 ^b^	12.70 ± 0.20 ^b^	13.26 ± 0.24 ^a,b^	13.77 ± 0.13 ^a^	0.005	0.58
Ash **	24.36 ± 0.22	20.58 ± 0.42 ^b^	21.33 ± 0.32 ^a,b^	22.33 ± 0.17 ^a^	22.71 ± 0.19 ^a^	0.005	0.57

^#^ The regressions were considered significant when *p* < 0.05. * On a fresh basis. ** On a dry matter basis. Data were expressed as Mean ± SE, n = 3; ^a,b,c^ Mean values in the same row with different superscripts differ significantly (*p* < 0.05).

**Table 4:** The effects of dietary doum palm fruit powder (DPFP) on the hematological and serum biochemical indices of African catfish.

Parameters	DPFP0	DPFP5	DPFP10	DPFP15	Regression Analysis ^#^	
					Linear	Quadratic
Hematological indices						
RBCs (10^6^ mL^−1^)	1.70 ± 0.01 ^c^	2.52 ± 0.06 ^b^	2.83 ± 0.07 ^a^	2.89 ± 0.02 ^a^	0.00	0.001
Hb (g dL^−1^)	6.15 ± 0.15 ^b^	9.05 ± 0.05 ^a^	9.55 ± 0.25 ^a^	10.00 ± 0.20 ^a^	0.00	0.003
Ht (%)	18.40 ± 0.30 ^c^	25.85 ± 0.55 ^b^	32.20 ± 2.20 ^a,b^	35.72 ± 0.48 ^a^	0.002	0.16
WBCs (10^3^ mL^−1^)	1.19 ± 0.70 ^b^	3.64 ± 0.28 ^a^	4.54 ± 0.60 ^a^	4.97 ± 0.50 ^a^	0.00	0.01
Biochemical indices						
Glucose (mg dL^−1^)	87.5 ± 0.5 ^a^	84.0 ± 1 ^ab^	79.5 ± 0.5 ^b^	77.35 ± 0.10 ^b^	0.002	0.06
Cholesterol (mg dL^−1^)	143.5 ± 1.5 ^a^	139.5 ± 0.5 ^a^	126.5 ± 1.5 ^b^	107.50 ± 2.5 ^c^	0.001	0.01
Triglycerides (mg dL^−1^)	147.0 ± 5 ^a^	128.5 ± 0.5 ^a,b^	104.5 ± 9.5 ^b^	100.0 ± 1.5 ^b^	0.00	0.26
Total protein (g dL^−1^)	6.28 ± 0.19 ^c^	7.48 ± 0.41 ^b,c^	8.46 ± 0.03 ^a,b^	9.36 ± 0.10 ^a^	0.00	0.23
Albumin (g dL^−1^)	2.89 ± 0.11 ^a^	2.86 ± 0.26 ^a^	2.94 ± 0.14 ^a^	3.09 ± 0.13 ^a^	0.43	0.63
Total globulin (g dL^−1^)	3.39 ± 0.08 ^d^	4.62 ± 0.15 ^c^	5.52 ± 0.10 ^b^	6.30 ± 0.10 ^a^	0.004	0.11
α globulin-1 (g dL^−1^)	0.72 ± 0.05 ^a^	0.72 ± 0.02 ^a^	0.71 ± 0.02 ^a^	0.73 ± 0.01 ^a^	0.94	0.88
α globulin-2 (g dL^−1^)	0.87 ± 0.03 ^a^	0.84 ± 0.03 ^a^	0.83 ± 0.06 ^a^	0.82 ± 0.05 ^a^	0.34	0.84
ß globulin (g dL^−1^)	1.23 ± 0.11 ^a^	1.24 ± 0.06 ^a^	1.29 ± 0.07 ^a^	1.30 ± 0.04 ^a^	0.52	0.95
ɣ globulin (g dL^−1^)	0.57 ± 0.17 ^c^	1.82 ± 0.14 ^b^	2.69 ± 0.05 ^a^	2.88 ± 0.02 ^a^	0.001	0.12

^#^ The regressions were considered significant when *p* < 0.05. RBCs, erythrocyte count; Hb, hemoglobin concentration; Ht, hematocrit; WBCs, leukocyte count. Data were expressed as Mean ± SE, n = 9; ^a,b,c^ Mean values in the same row with different superscripts differ significantly (*p* < 0.05).

**Table 5:** The effects of dietary doum palm fruit powder (DPF) on the hepatic antioxidant capacity and immunological indices of African catfish.

Parameters	DPFP0	DPFP5	DPFP10	DPFP15	Regression Analysis ^#^	
					Linear	Quadratic
Antioxidant capacity						
CAT (U g^−1^ tissue)	1.84 ± 0.06 ^c^	2.86 ± 0.11 ^b^	3.34 ± 0.09 ^b^	3.97 ± 0.13 ^a^	0.001	0.98
SOD (U g^−1^ tissue)	4.7 ± 0.36 ^b^	11.6 ± 1.61 ^b^	19.85 ± 1.05 ^a^	22.87 ± 1.63 ^a^	0.001	0.20
GSH (mmol g^−^^1^ tissue)	1.58 ± 0.49 ^b^	1.63 ± 0.29 ^b^	3.36 ± 0.06 ^a^	4.64 ± 0.12 ^a^	0.00	0.10
Immunological indices						
Phagocytic%	55.50 ± 0.50 ^c^	66.00 ± 0.82 ^b^	70.50 ± 1.01 ^a^	80.50 ± 0.55 ^a^	0.003	0.72
Phagocytic index	2.75 ± 0.25 ^b^	3.85 ± 0.05 ^a^	4.60 ± 0.10 ^a^	5.25 ± 0.50 ^a^	0.004	0.18
Lysozyme (µg mL^−1^)	14.59 ± 0.8 ^c^	23.7 ± 1.71 ^b^	30.61 ± 0.61 ^a^	39.50 ± 0.45 ^a^	0.001	0.92
NO (µmol L^−1^)	52.98 ± 1.11 ^c^	75.6 ± 3.04 ^b^	93.27 ± 2.18 ^a^	99.50 ± 0.50 ^a^	0.001	0.01
MPO (U L^−1^)	101.01 ± 3 ^a^	85.00 ± 2.00 ^a^	65.02 ± 4.5 ^b^	57.00 ± 2.01 ^b^	0.00	0.28
α 2,3-ST	0.05 ± 0.01 ^c^	0.58 ± 0.02 ^b^	0.67 ± 0.01 ^a^	0.75 ± 0.02 ^a^	0.02	0.23
α 2,6-ST	0.14 ± 0.02 ^c^	0.69 ± 0.01 ^b^	0.92 ± 0.02 ^a^	0.98 ± 0.01 ^a^	0.01	0.15

^#^ The regressions were considered significant at *p* < 0.05. CAT, catalase; SOD, superoxide dismutase; GSH, reduced glutathione; NO, nitric oxide; MPO, myeloperoxidase; α 2,3-ST, α 2,3-sialyltransferase; α 2,6-ST, α 2,6-sialyltransferase. Data were expressed as Mean ± SE, n = 9; ^a,b,c^ Mean values in the same row with different superscripts differ significantly (*p* < 0.05).

## References

[1] FAO (2018). The State of World Fisheries and Aquaculture 2018-Meeting the Sustainable Development Goals.

[2] Adeshina I., Abdel-Tawwab M. (2020). Dietary taurine incorporation to high plant protein-based diets improved growth, biochemical, immunity, and antioxidants biomarkers of African catfish, *Clarias gariepinus* (B.). Fish Physiol. Biochem..

[3] Kemigabo C., Abdel-Tawwab M., Lazaro J.W., Sikawa D., Masembe C., Kang’Ombe J. (2018). Combined effect of dietary protein and phytase levels on growth performance, feed utilization, and nutrients digestibility of African catfish, *Clarias gariepinus* (B.), reared in earthen ponds. J. Appl. Aquacult..

[4] Dadebo E. (2000). Reproductive biology and feeding habits of the catfish Clarias gariepinus (Burchell) (Pisces: Clariidae) in Lake Awassa, Ethiopia. SINET Ethiop. J. Sci..

[5] Idodo-Umeh G. (2003). Fresh Water Fishes of Nigeria. Taxonomy, Ecological Notes, Diet and Utilization.

[6] El-Araby D.A., Amer S.A., Khalil A.A. (2020). Effect of different feeding regimes on the growth performance, antioxidant activity, and health of Nile tilapia, *Oreochromis niloticus*. Aquaculture.

[7] Dada A.A. (2017). Use of fluted pumpkin (Telfairia occidentalis) leaf powder as feed additive in African catfish (*Clarias gariepinus*) fingerlings. J. Appl. Anim. Res..

[8] Bostock J., McAndrew B., Richards R., Jauncey K., Telfer T., Lorenzen K., Little D., Ross L., Handisyde N., Gatward I. (2010). Aquaculture: Global status and trends. Philos. Trans. R. Soc. B Biol. Sci..

[9] Mokoro A., Oyoo-Okoth E., Ngugi C.C., Njiru J., Rasowo J., Chepkirui-Boit V., Manguya-Lusega D. (2014). Effects of stocking density and feeding duration in cage-cum-pond-integrated system on growth performance, water quality and economic benefits of *Labeo victorianus* (Boulenger 1901) culture. Aquacult. Res..

[10] Ashiru A., Uaboi-Egbeni P., Oguntowo J., Idika C. (2011). Isolation and antibiotic profile of *Aeromonas* species from tilapia fish (Tilapia nilotica) and catfish (*Clarias betrachus*). Pak. J. Nutr..

[11] Zhang X., Yang W., Wu H., Gong X., Li A. (2014). Multilocus sequence typing revealed a clonal lineage of *Aeromonas hydrophila* caused motile *Aeromonas* septicemia outbreaks in pond-cultured cyprinid fish in an epidemic area in central China. Aquaculture.

[12] Romero J., Feijoó C.G., Navarrete P. (2012). Antibiotics in aquaculture–Use, abuse and alternatives. Health Environment in Aquaculture.

[13] Cabello F.C. (2006). Heavy use of prophylactic antibiotics in aquaculture: A growing problem for human and animal health and for the environment. Environ. Microbiol..

[14] Bharathi S., Antony C., Cbt R., Arumugam U., Ahilan B., Aanand S. (2019). Functional feed additives used in fish feeds. Int. J. Fish. Aquat. Stud..

[15] Abdel Rahman A.N., ElHady M., Hassanin M.E. (2018). Effect of Indian lotus (*Nelumbo nucifera Gaertn.*) leaf powder on immune status and disease resistance of Nile tilapia. Aquacult. Res..

[16] Abdel Rahman A.N., Khalil A.A., Abdallah H., ElHady M. (2018). The effects of the dietary supplementation of Echinacea purpurea extract and/or vitamin C on the intestinal histomorphology, phagocytic activity, and gene expression of the Nile tilapia. Fish Shellfish Immunol..

[17] Amer S.A., Ahmed S.A., Ibrahim R.E., Al-Gabri N.A., Osman A., Sitohy M. (2020). Impact of partial substitution of fish meal by methylated soy protein isolates on the nutritional, immunological, and health aspects of Nile tilapia, *Oreochromis niloticus* fingerlings. Aquaculture.

[18] Amer S.A., Metwally A.E., Ahmed S.A. (2018). The influence of dietary supplementation of cinnamaldehyde and thymol on the growth performance, immunity and antioxidant status of monosex Nile tilapia fingerlings (*Oreochromis niloticus*). Egypt. J. Aquat. Res..

[19] Abdel Rahman A.N., ElHady M., Shalaby S.I. (2019). Efficacy of the dehydrated lemon peels on the immunity, enzymatic antioxidant capacity and growth of Nile tilapia (*Oreochromis niloticus*) and African catfish (*Clarias gariepinus*). Aquaculture.

[20] Aboshora W., Lianfu Z., Dahir M., Gasmalla M.A., Musa A., Omer E., Thapa M. (2014). Physicochemical, nutritional and functional properties of the epicarp, flesh and pitted sample of doum fruit (*Hyphaene Thebaica)*. J. Food Nutr. Res..

[21] El-Beltagi H.S., Mohamed H.I., Yousef H.N., Fawzi E.M. (2018). Biological Activities of the Doum Palm (*Hyphaene thebaica* L.) Extract and Its Bioactive Components. Antioxidants in Foods and Its Applications.

[22] Hussein A.M., Salah Z.A., Hegazy N.A. (2010). Physicochemical, sensory and functional properties of wheat-doum fruit flour composite cakes. Pol. J. Food Nutr. Sci..

[23] Siddeeg A., Salih Z., Al-Farga A., Ata-Elfadeel E., Ali A. (2019). Physiochemical, Nutritional and Functional Properties of Doum (*Hyphene thebaica*) Powder and Its Application in Some Processed Food Products. J Nutr. Food Sci. Forecast.

[24] Bayad A.E. (2016). Influences of doum fruit *Hyphaene thebaica* extract on the reproductive parameters, blood picture, lipid profile and hepato-renal functions in rats. MRJMMS.

[25] Hoseinifar S.H., Dadar M., Khalili M., Cerezuela R., Esteban M.Á. (2017). Effect of dietary supplementation of palm fruit extracts on the transcriptomes of growth, antioxidant enzyme and immune-related genes in common carp (*Cyprinus carpio*) fingerlings. Aquacult. Res..

[26] Hoseinifar S.H., Khalili M., Rufchaei R., Raeisi M., Attar M., Cordero H., Esteban M.Á. (2015). Effects of date palm fruit extracts on skin mucosal immunity, immune related genes expression and growth performance of common carp (*Cyprinus carpio*) fry. Fish Shellfish Immunol..

[27] Cerezuela R., Guardiola F.A., Cuesta A., Esteban M.Á. (2016). Enrichment of gilthead seabream (*Sparus aurata* L.) diet with palm fruit extracts and probiotics: Effects on skin mucosal immunity. Fish Shellfish Immunol..

[28] Guardiola F., Porcino C., Cerezuela R., Cuesta A., Faggio C., Esteban M. (2016). Impact of date palm fruits extracts and probiotic enriched diet on antioxidant status, innate immune response and immune-related gene expression of European seabass (*Dicentrarchus labrax*). Fish Shellfish Immunol..

[29] AOAC (2000). Official Methods of Analysis of AOAC International.

[30] Council N.R. (2011). Nutrient Requirements of Fish and Shrimp.

[31] CCAC (2005). Canadian Council on Animal Care Guidelines on: The Care and Use of Fish in Research, Teaching and Testing.

[32] APHA (1998). Water Environment Federation 1998. Standard Methods for the Examination of Water and Wastewater.

[33] Jayant M., Hassan M., Srivastava P., Meena D., Kumar P., Kumar A., Wagde M. (2018). Brewer’s spent grains (BSGs) as feedstuff for striped catfish, *Pangasianodon hypophthalmus* fingerlings: An approach to transform waste into wealth. J. Clean. Prod..

[34] Adeshina I., Jenyo-Oni A., Emikpe B. (2016). Use of eugenia cayrophyllata oil as anaesthetic in farm raised african catfish *clarias gariepinus* juveniles. Egypt. J. Exp. Biol. (Zool.).

[35] Suvarna S., Layton C., Bancroft J. (2013). The Hematoxylins and Eosin. Bancroft’s Theory and Practice of Histological Techniques.

[36] Pirarat N., Pinpimai K., Endo M., Katagiri T., Ponpornpisit A., Chansue N., Maita M. (2011). Modulation of intestinal morphology and immunity in nile tilapia (*Oreochromis niloticus*) by Lactobacillus rhamnosus GG. Res. Vet. Sci..

[37] Feldman B., Zinkl J., Jain N.C., Schalm O.W. (2000). Schalm’s Veterinary Hematology.

[38] Jain N.C. (1993). Essentials of Veterinary Hematology.

[39] Allain C.C., Poon L.S., Chan C.S., Richmond W., Fu P.C. (1974). Enzymatic determination of total serum cholesterol. Clin. Chem..

[40] McGowan M., Artiss J.D., Strandbergh D.R., Zak B. (1983). A peroxidase-coupled method for the colorimetric determination of serum triglycerides. Clin. Chem..

[41] Trinder P. (1969). Determination of blood glucose using 4-amino phenazone as oxygen acceptor. J. Clin. Pathol..

[42] Kaplan A., Savory J. (1965). Evaluation of a cellulose-acetate electrophoresis system for serum protein fractionation. Clin. Chem..

[43] Sinha A.K. (1972). Colorimetric assay of catalase. Anal. Biochem..

[44] McCord J.M., Fridovich I. (1969). Superoxide dismutase an enzymic function for erythrocuprein (hemocuprein). J. Biol. Chem..

[45] Patterson J. (1955). Determination of glutathione. Methods Biochem. Anal..

[46] Siwicki A.K., Anderson D.P., Rumsey G.L. (1994). Dietary intake of immunostimulants by rainbow trout affects non-specific immunity and protection against furunculosis. Vet. Immunol. Immunopathol..

[47] Grinde B. (1989). Lysozyme from rainbow trout, Salmo gairdneri Richardson, as an antibacterial agent against fish pathogens. J. Fish Dis..

[48] Quade M.J., Roth J.A. (1997). A rapid, direct assay to measure degranulation of bovine neutrophil primary granules. Vet. Immunol. Immunopathol..

[49] Moshage H. (2009). Simple and reliable measurement of nitric oxide metabolites in plasma. Clin. Chem..

[50] Kletter D., Singh S., Bern M., Haab B.B. (2013). Global comparisons of lectin–glycan interactions using a database of analyzed glycan array data. Mol. Cell. Proteom..

[51] Zhou F., Song W., Shao Q., Peng X., Xiao J., Hua Y., Owari B.N., Zhang T., Ng W.K. (2011). Partial replacement of fish meal by fermented soybean meal in diets for black sea bream, *Acanthopagrus schlegelii*, juveniles. J. World Aquacult. Soc..

[52] Scheidegger E., Fracalanzza S., Teixeira L., Cardarelli-Leite P. (2009). RFLP analysis of a PCR-amplified fragment of the 16S rRNA gene as a tool to identify Enterococcus strains. Mem. Inst. Oswaldo Cruz.

[53] Lucky Z. (1977). Methods for the Diagnosis of Fish Diseases.

[54] Amend D.F. (1981). Potency testing of fish vaccines. Fish Biologics: Serodiagnostics and Vaccines.

[55] Burkill H.M. (1995). The Useful Plants of West Tropical AFRICA.

[56] Nwosu F., Dosumu O., Okocha J. (2008). The potential of *Terminalia catappa* (Almond) and *Hyphaene thebaica* (Dum palm) fruits as raw materials for livestock feed. Afr. J. Biotechnol..

[57] Chakraborty S.B., Horn P., Hancz C. (2014). Application of phytochemicals as growth-promoters and endocrine modulators in fish culture. Rev. Aquacult..

[58] Pirarat N., Boonananthanasarn S., Krongpong L., Katagiri T., Maita M. (2015). Effect of activated charcoal-supplemented diet on growth performance and intestinal morphology of Nile tilapia (*Oreochromis niloticus*). Thai J. Vet. Med..

[59] Amer S.A., Osman A., Al-Gabri N.A., Elsayed S.A., Abd El-Rahman G.I., Elabbasy M.T., Ahmed S.A., Ibrahim R.E. (2019). The Effect of Dietary Replacement of Fish Meal with Whey Protein Concentrate on the Growth Performance, Fish Health, and Immune Status of Nile Tilapia Fingerlings, *Oreochromis niloticus*. Animals.

[60] Hershberg R., Blumberg R.S. (2003). The lymphocyte-epithelial-bacterial interface. Inflammatory Bowel Disease: From Bench to Bedside.

[61] Munglue P. (2016). Effects of lotus (Nelumbo nucifera Gaertn.) stamen extract on growth performance, feed utilization and intestinal morphology of catfish (*Clarias gariepinus*). Asia Pac. J. Sci. Technol..

[62] Abdel-Tawwab M., Adeshina I., Jenyo-Oni A., Ajani E.K., Emikpe B.O. (2018). Growth, physiological, antioxidants, and immune response of African catfish, *Clarias gariepinus* (B.), to dietary clove basil, Ocimum gratissimum, leaf extract and its susceptibility to Listeria monocytogenes infection. Fish Shellfish Immunol..

[63] Orire A. (2010). Protein Sparing Effects of Carbohydrate and Lipid in the Practical Diets of Catfish (Clarias gariepinus) and Tilapia (*Oreochromis niloticus*) Production. Ph.D. Thesis.

[64] Solomon R., Oluchi A. (2018). Proximate Analysis and Nutritional Value of African Catfish (Clarias gariepinus) Fed with Local (Telferia occidentales and Moringa olefera) and Industrial Feed (Coppens). J. Fish. Livest. Prod..

[65] Satheeshkumar P., Senthilkumar D., Ananthan G., Soundarapandian P., Khan A.B. (2011). Measurement of hematological and biochemical studies on wild marine carnivorous fishes from Vellar estuary, southeast coast of India. Comp. Clin. Pathol..

[66] Arup T., Patra B. (2011). Oral administration of baker’s yeast (*Saccharomyces cerevisiae*) acts as a growth promoter and immunomodulator in Labeo rohita (Ham.). J. Aquacult. Res. Dev..

[67] Amer S.A. (2016). Effect of Spirulina platensis as feed supplement on growth performance, immune response and antioxidant status of mono-sex Nile Tilapia (*Oreochromis niloticus*). Benha Vet. Med. J..

[68] Kamis A., Modu S., Zanna H., Oniyangi T. (2003). Preliminary biochemical and haematological effects of aqueous suspension of pulp of *hyphaene thebaica* (l) mart in rats. Biokemistri.

[69] Auwal M., Sanda K., Mairiga I., Lawan F., Mutah A., Tijjani A., Shuaibu A., Ibrahim A., Njobdi A., Thaluvwa A. (2013). The Phytochemical, Elemental and Hematologic Evaluation of Crude Mesocarp Extract of *Hyphaene thebaica* (doumpalm) in Wistar Albino Rats. Asian J. Biochem..

[70] Whyte S.K. (2007). The innate immune response of finfish–a review of current knowledge. Fish Shellfish Immunol..

[71] Adegbesan S.I., Obasa S.O., Abdulraheem I. (2018). Growth performance, haematology and histopathology of African catfish (*Clarias gariepinus*) fed varying levels of Aloe barbadensis leaves. J. Fish..

[72] Olusola S.E., Nwokike C.C. (2018). Effects of dietary leaves extracts of bitter (Vernonia amygdalina) and pawpaw (Carica papaya) on the growth, feed conversion efficiency and disease resistance on juveniles Clarias gariepinus. Aquacult. Res..

[73] Abdel-Rahim E.A., El-Beltagi H.S., Fayed S.A. (2011). Comparative studies on the influences of Juniperus phoenicea and Hyphaena thebaica as hypoglycemic factors in diabetic rats. Adv. Food Sci..

[74] Hetta M., Yassin N. (2006). Comparative studies on hypocholesterolemic effect of different fractions of *Hyphaene thebaica* (Doum) in experimental animals. Pharm. Int. J. Pharm. Sci..

[75] Mohamed A.A., Khalil A.A., El-Beltagi H.E. (2010). Antioxidant and antimicrobial properties of kaff maryam (*Anastatica hierochuntica*) and doum palm (Hyphaene thebaica). Grasas Aceites.

[76] Gabriel N.N., Wilhelm M.R., Habte-Tsion H.-M., Chimwamurombe P., Omoregie E., Iipinge L.N., Shimooshili K. (2019). Effect of dietary Aloe vera polysaccharides supplementation on growth performance, feed utilization, hemato-biochemical parameters, and survival at low pH in African catfish (*Clarias gariepinus*) fingerlings. Int. Aquat. Res..

[77] Asadi M., Mirvaghefei A., Nematollahi M., Banaee M., Ahmadi K. (2012). Effects of Watercress (Nasturtium nasturtium) extract on selected immunological parameters of rainbow trout (*Oncorhynchus mykiss*). Open Vet. J..

[78] Iheanacho S., Ogueji E., Yaji A., Dada O., Mbah C., Ifejimalu A., Ibrahim B.-U. (2017). Effects of herbal plants (*Zingiber officinale and Hibiscus sabdariffa*) as dietary additives on serum biochemistry and some metabolites in *Clarias gariepinus* (Burchell, 1822). J. Coast. Life Med..

[79] Saglam D., Atli G., Dogan Z., Baysoy E., Gurler C., Eroglu A., Canli M. (2014). Response of the antioxidant system of freshwater fish (*Oreochromis niloticus*) exposed to metals (Cd, Cu) in differing hardness. Turk. J. Fish. Aquat. Sci..

[80] Kornsteiner M., Wagner K.-H., Elmadfa I. (2006). Tocopherols and total phenolics in 10 different nut types. Food Chem..

[81] Ayoub N.A., Eldahshan O.A., Singab A.-N.B., Al-Azizi M.M. (2011). Chemical composition of essential oil from doum fruits Hyphaene thebaica (Palmae). J. Essent. Oil Bear. Plants.

[82] Esteban M., Cordero H., Martínez-Tomé M., Jiménez-Monreal A., Bakhrouf A., Mahdhi A. (2014). Effect of dietary supplementation of probiotics and palm fruits extracts on the antioxidant enzyme gene expression in the mucosae of gilthead seabream (*Sparus aurata* L.). Fish Shellfish Immunol..

[83] Uribe C., Folch H., Enríquez R., Moran G. (2011). Innate and adaptive immunity in teleost fish: A review. Vet. Med..

[84] Neumann N.F., Stafford J.L., Barreda D., Ainsworth A.J., Belosevic M. (2001). Antimicrobial mechanisms of fish phagocytes and their role in host defense. Dev. Comp. Immunol..

[85] Magnadóttir B., Lange S., Steinarsson A., Gudmundsdottir S. (1999). Infection dynamics of two renal myxozoans in hatchery reared fry and juvenile Atlantic cod *Gadus morhua* L.. Comp. Biochem. Physiol. B Biochem. Mol. Biol..

[86] Vasta G.R., Ahmed H., Nita-Lazar M., Banerjee A., Pasek M., Shridhar S., Guha P., Fernández-Robledo J.A. (2012). Galectins as self/non-self recognition receptors in innate and adaptive immunity: An unresolved paradox. Front. Immunol..

[87] Coombe D.R., Parish C.R. (2015). Carbohydrates: The yet to be tasted sweet spot of immunity. Front. Immunol..

[88] Aratani Y. (2018). Myeloperoxidase: Its role for host defense, inflammation, and neutrophil function. Arch. Biochem. Biophys..

[89] Farag M.A., Paré P.W. (2013). Phytochemical Analysis and Anti-inflammatory Potential of *Hyphaene thebaica* L. Fruit. J. Food Sci..

[90] Köllner B., Wasserrab B., Kotterba G., Fischer U. (2002). Evaluation of immune functions of rainbow trout (*Oncorhynchus mykiss*)—How can environmental influences be detected?. Toxicol. Lett..

[91] Sakai M., Taniguchi K., Mamoto K., Ogawa H., Tabata M. (2001). Immunostimulant effects of nucleotide isolated from yeast RNA on carp, Cyprinus carpio L.. J. Fish Dis..

